# The Influence of Music Preference on Exercise Responses and Performance: A Review

**DOI:** 10.3390/jfmk6020033

**Published:** 2021-04-08

**Authors:** Christopher G. Ballmann

**Affiliations:** Department of Kinesiology, Samford University, Birmingham, AL 35229, USA; cballman@samford.edu

**Keywords:** preferred music, non-preferred music, exercise training, resistance exercise

## Abstract

Listening to music has been repeatedly shown to have ergogenic benefits during various modes of exercise, including endurance, sprint, and resistance-based activities. Music is commonly incorporated into training regimens by recreational exercisers and competitive athletes alike. While specific modalities of exercise elicit varying physiological responses, listening to music has been shown to modulate many of these responses (i.e., heart rate, catecholamines, muscle activation) often leading to improved performance. Furthermore, listening to music during exercise may positively impact psychological (i.e., mood, motivation) and psychophysiological (i.e., rate of perceived exertion, arousal) changes, which may allow for favorable responses during an exercise challenge. However, there is mixed evidence regarding music’s efficacy, which may be mediated through differences in music selection and preference. Emerging evidence has shown that, whether an individual prefers or does not prefer the music they are listening to during exercise greatly influences their ergogenic potential in addition to physiological, psychological, and psychophysiological responses to exercise. From a practical standpoint, music may be controlled by the individual through headphones but is often played communally over speakers in locker rooms, gyms, and health clubs, which may have consequences on performance and training. The following review will describe the physiological, psychological, and psychophysiological responses to exercise while listening to music and how music preference may particularly alter them. Current knowledge and new evidence on how music preference factors into enhancing performance in various modes of exercise will be further discussed, incorporating practical considerations for individuals and practitioners in real-world applications to optimize performance.

## 1. Introduction

Descriptions of listening to different types of rhythms and melodies during competition or battle have been noted to go back thousands of years [1]. Even centuries ago, different types of music often had the power to propel individuals to conflict or peace between cultures and religions [2]. Indeed, early emphasis on the importance of music preference has been described to affect individuals’ overall feelings of positivity or negativity [3]. As 19th century composer Thomas Surrette stated, “For if it is bad music, the more we hear it the worse off we are”. Thus, the individual and cultural effects of music preference have been widely described, but the distinct role it has in optimizing modern-day sport and exercise performance has only recently become a prominent topic of investigation.

Music during competition became particularly prominent during the 20th century Olympic competitions. Between Olympic ceremonies (i.e., opening, medal awards) and incorporation into some events (i.e., gymnastics, figure skating), the association of music and sports became more widely accepted [4]. Currently, music is associated with almost all sporting events and has been suggested as one of the primary phenomenon with sport competition [5]. With the rise of recreational and individualized exercise programs, incorporation of music into training has increased. New developments in portable technology (i.e., smart phones, mp3 players) have increased ease of access and allowed for individual choice of music during exercise [6]. Elite athletes have reported listening to music during exercise training sessions, pre-competition, and warm up on the basis of their belief that it improves mood, motivation, and aids in achieving top performance levels [7]. With this, large amounts of investigations have focused on the potential benefits of music during exercise primarily as, but not limited to, a means for improving peak performance.

Listening to music has been shown to improve performance in endurance [8,9], sprint [10,11], and resistance modes of exercise [12,13,14]. The ergogenic and performance-enhancing effects of music may be achieved through several different alterations to the exercise response. Music has been shown to potently reduce the perception of fatigue and exertion through dissociation and distraction during exercise [15,16]. Increases in arousal and neural activity while listening to music have been shown to accompany improved exercise performance [17,18]. The synchronization of music and exercise may result in improved running economy, efficiency, and overall performance [19,20,21]. Listening to music prior to and during exercise has been shown to increase motivation and effort, leading to improved performance outcomes [9,12,14]. Improvements in performance may also be mediated through improved mood, exercise enjoyment, and increased feelings of power [10,18,22]. Thus, the effects of music on exercise performance are multi-faceted, allowing for possible benefits in a wide arrange of athletic populations and exercise modalities. However, it should be noted that mixed results exist with some studies, showing little to no benefits of listening to music on performance [16,23,24]. While distinct factors for disparities in findings are not fully clear, recent evidence from my lab and others, showing that music selection and preference may largely mediate the ergogenic potential of music, suggests a principal importance in music choice when identifying the possible benefits of music. Thus, the primary focus of this review is to provide existing and novel evidence on how music preference may optimize performance.

Music and performance have been systematically explored, amounting to a wealth of evidence describing effects and/or benefits during exercise and training. Due to this, not all literature can be encompassed in the current review. For a more comprehensive literature review, the reader is directed to the following review by Karageorghis and colleagues [25]. The following review will primarily focus on the role that music preference plays in relation to exercise responses and performance. A general background on how music influences the different types of exercise responses will be discussed, followed by how music selection and preference are commonly determined. The remainder of the review will describe evidence for how music preference may mediate exercise responses and performance benefits while identifying which factors may be particularly beneficial to optimizing performance. Finally, practical implications for exercisers and practitioners will be highlighted with considerations for strategies to elicit peak performance through music preference.

## 2. General Exercise Responses While Listening to Music

**Figure 1:**
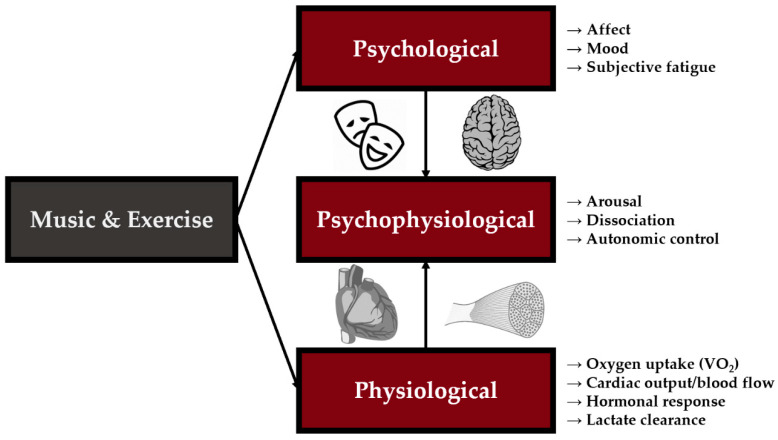
Three primary areas music has been suggested to have the largest impact on are: psychological, physiological, and psychophysiological. Selected variables by which music alters the experience response are shown although this is not an exhaustive list. Ultimately, psychological and physiological factors influence one another, creating an interrelated archetype for changes during exercise while listening to music.

Music has been postulated to influence exercise performance through three main types of mechanisms: psychological, physiological, and psychophysiological (Figure 1) [26]. The following two sections will briefly review the psychological and physiological changes to exercise while listening to music. Additionally, intertwined within each of these sections is the interrelatedness of these areas via psychophysiological mechanisms. These sections are in place to serve as a foundation for explaining mechanistic findings of how music preference may influence exercise responses in later sections.

### 2.1. Psychological

Psychological changes with exercise have been widely described to influence sport and exercise performance [27]. Psychological responses influencing exercise performance may be related to well-being, cognitive, emotional, and behavioral domains which may impact both exercise compliance and capacity [28]. For example, lower tension, depression, anger, and higher vigor have been associated with more successful athletic performance [29]. Independent of exercise, music has been suggested to influence multiple psychological domains [30]. Indeed, previous evidence has reported improvements in positive feelings, mood, and subjective fatigue while listening to music [31]. Thus, the reliance of favorable exercise performance on various psychological states and the abilities of music to alter them has resulted in comprehensive research investigating the psychological influences of music on peak performance. While the literature presented in this section does not encompass all psychological responses to exercise, key aspects which may be highly applicable to music and exercise performance are discussed.

Interrelated emotional and mood states are commonly measured during exercise utilizing the Profile of Mood States (POMS) questionnaire or other tools which encompass the specific areas of anxiety, anger, vigor, fatigue, depression, and confusion or the Feelings Scale, which quantifies valence and feelings into “good/positive” or “bad/negative” [32,33]. Affective valence refers to the positivity/negativity or goodness/badness an individual perceives intrinsically about a particular factor or thing [34]. Positive valence has been shown to acutely increase with both endurance and resistance-based exercise but may be to a greater degree with higher intensity activity [35,36]. With music and exercise combined, Hutchison et al. showed that individuals were able to maintain exercise at a greater intensity while still maintaining a “good” feeling when listening to self-selected music versus when they listened to no music [37]. Bolstering this, Elliot et al. reported that listening to motivational music resulted in greater workloads completed during cycling with concomitant improvements in positive affective valence [38]. Collectively, these findings, among others suggest that positive affect can be induced while listening to music during exercise despite higher intensity and workloads. Positive affect during vigorous exercise has also been shown in untrained individuals during graded treadmill exercise [39]. Improvements in affective responses during exercise have additionally been shown to be increased while listening to music in exercise resistant populations [40]. Thus, improvements in affective responses during exercise while listening to music are not just limited to well-trained athletes and may apply to broader populations looking to improve performance and physical activity levels.

Vigor, or subjective feelings of energy and vitality, have been shown to be increased during exercise while listening to music [41]. Chtourou et al. showed that listening to music during a warm-up increased vigor in sprinters, resulting in improved power output and anaerobic performance [42]. Furthermore, Biagani et al. showed that listening to self-selected music increased vigor in resistance trained men and rate of velocity and force development of ballistic jumping, albeit bench press performance was largely unaffected [18]. Vigor may also be paired with physiological changes, resulting in the psychophysiological phenomena of power and arousal. Hsu et al. reported that listening to music increased subjective feelings of power which may be dependent on inherent characteristics of music [22]. These feelings may also mediate determination of whether an individual responds favorably or prefers a particular type of music. Optimal arousal levels have been implicated in peak exercise performance and follow an inverted “u-shape” pattern, whereby too much or too little arousal results in performance decrements [43]. Music has been well described to mediate arousal during exercise [44]. Indeed, inherent characteristics of music, such as high tempo and volume, have been implicated to mediate increases in arousal with concomitant increases in performance [45]. Changes in arousal while listening to music are not fully understood, but have been shown in imaging studies to manifest in particular brain regions involved in emotion and affective responses. Bigilassi et al. reported increases in left inferior frontal gyrus activity, which was suggested to upregulate arousal and divert attention away from the exercise stimulus [46]. Thus, affective arousal changes while listening to music appear to also have physiological underpinnings, which taken together may mediate ergogenic benefits.

Contrasting to vigor and arousal, subjective fatigue, or the feeling of a lack of energy, has also been shown to be modulated positively by music. Liu et al. reported that fast tempo music interventions delay sports and exercise-related mental fatigue [47]. Improvements in fatigue may also be related to exercise recovery. Listening to relaxing music following exercise has been shown to improve recovery and attenuate perceived fatigue [48]. Related to fatigue, the perception of exertion (RPE) has perhaps been the most consistent factor which has been reported to be altered during exercise by music. For example, Nakamura et al. showed that RPE was significantly lower while listening to music during endurance cycling [8]. Music-mediated lowering of RPE has also been shown in other modes of exercise, including high intensity repeated sprints and resistance exercise [16,49]. Lower RPE is likely due to dissociation, whereby the external stimulus of music distracts or diverts attention away from discomfort and giving effort [50]. The physiological basis for lower RPE has been described whereby brain activity is altered due to the reallocation of attention to the external stimulus of exercise [46]. However, it should be mentioned that, for each of the previously discussed psychological factors, a number of investigations have shown little to no changes during exercise or shown no influence on performance. While the cause of disparities between findings is not fully clear, differences may be due to differing modes of exercise, intensity, participant population, and music type and selection. Whether an individual prefers or does not prefer the music they are listening to has been shown to potently influence mood states and RPE [16,51]. Thus, the consideration of preference while listening to music during exercise may be especially important.

### 2.2. Physiological

Physiological mechanisms that are responsible for performance enhancement while listening to music are numerous but precise contributions are difficult to isolate. In part, this may be due to concomitant systemic physiological changes during exercise in addition to the pleiotropic nature of the response to music. The collective evidence suggests that music induces alterations in physiology in two broad areas: (1) neural activation (i.e., brain activity, autonomic responses, etc.), (2) metabolic responses (i.e., VO_2_/energy expenditure, lactate clearance, hypothalamic pituitary axis control, etc.). While other evidence outside of these areas has been documented, these appear to be the most relevant to the exercise response and will be the focus of the remaining portion of this section.

Although conflicting evidence exists, a multitude of investigations, many of which cannot be covered in this review, have described music induced neural changes during exercise both centrally and peripherally. Multiple investigations have shown that music increases activity in portions of the brain that are important for physiological arousal, emotion, and perception [46]. Previous studies have reported increases in left inferior frontal gyrus and insular cortex activation while listening to music during isometric exercise using functional magnetic resonance imaging (fMRI) [46]. The activation of these brain regions may suggest possible increases in cognitive processing speed and the organization of movement while listening to music during exercise. The organization of movement with music may be particularly beneficial for exercise efficiency [21]. Furthermore, other evidence has shown theta wave activity, which reflects that relaxation and sleep, are decreased in the cortex surface while listening to music likely indicating changes in physiological arousal which may improve exercise performance [52]. Neurological changes while listening to music may also manifest themselves in peripheral divisions, such as autonomic and somatic nervous system activation. For example, listening to music during cycling has been linked to the prevention of decreased heart rate variability (HRV) following exercise indicating the preservation of parasympathetic stimulation following physical stress [53]. However, this may be differentially affected by the type of music the individual is listening to. Relaxing music has been shown to lower norepinephrine levels while fast tempo music has been shown to increase epinephrine levels with exercise [54]. Lower plasma catecholamines have been noted by other groups while listening to classical music during treadmill running [55]. While not confirmed, it has been suggested that the decreases in catecholamines reflect lower sympathetic output, thus possibly influencing blood and oxygen delivery to peripheral skeletal muscle [56]. Conversely, listening to arousing music during a warm-up has been shown to increase catecholamines, which may ultimately influence muscle activation and metabolic responses during subsequent exercise [57]. Regarding somatic motor activation, auditory stimuli have been reported to be coupled to motor control and output. Rodriguez-Fornells et al. showed increased bilateral activation of the primary motor cortex and supplementary motor area when individuals listen to music during ambulation [58]. These changes resulted in improved upper body motor performance. Recently, Centala et al. showed that listening to fast tempo music increased the neuromuscular fatigue threshold during knee extensor exercise [59]. Importantly, reductions in neuromuscular fatigue occurred simultaneously to increased power output and performance, suggesting improved muscle efficiency [59]. Collectively, how music influences neurological factors during exercise is still being explicated, but current evidence suggests a unique ability for music to control autonomic stimulation during and following exercise, along with improved motor output performance.

Music has been well established to influence exercise metabolism indirectly. Listening to fast music has been shown to increase cardiac output and oxygen consumption (VO_2_) during steady-state exercise compared to no music [60]. Interestingly, this was accompanied by decreases in systemic vascular resistance. These findings would indicate that music may decrease cardiovascular efficiency, which could be deemed as undesirable during steady-state exercise. However, the simultaneous decreases in vascular resistance may also indicate that the increased cardiac output is paired with less impedance to blood flow which could be favorable during maximal exercise, as oxygen delivery can be a limiting factor to maximal performance [61]. This is further bolstered by findings of increased VO_2max_ while listening to fast tempo music during maximal aerobic exercise [62]. This plausible increase in blood flow may also partially explain previous findings of increased lactate clearance while listening to music. Ghaderi et al. showed that trained handball players had lower blood lactate levels following high-intensity exercise while listening to motivational music compared to no music [63]. Listening to motivational music during exercise recovery has also been shown to be associated with increased lactate clearance in active males [64]. If suggestions of increased blood flow to working skeletal muscle while listening to music hold true, then improvements in acute recovery may underpin ergogenic effects of music especially in the context of repeated bouts of exercise.

Listening to music during exercise has also been reported to alter hormonal responses, specifically in the hypothalamic pituitary axis (HPA). Brownley et al. showed that listening to fast tempo music during high intensity exercise resulted in higher salivary concentrations of cortisol compared to no music [65]. These findings have been reinforced by others showing that motivational music results in sustained elevations of cortisol following exercise [65]. While the consequences of altered cortisol responses on performance while listening to music are not fully clear, increased cortisol could improve substrate availability during exercise and recovery though increased gluconeogenesis and free fatty acid mobilization [66]. However, the effects of music on the HPA appears to be dependent on whether the music is considered sedative or stimulative. Sedative music has been shown to decrease cortisol levels following short-term high-intensity exercise [67]. Thus, this supports the idea that different types of music may alter physiological responses to exercise differently, further highlighting the importance of understanding how music preference influences exercise responses.

## 3. Music Preference and Exercise Performance

A broad working definition of preferred music is any song, genre, rhythm, etc., which individuals deem most favorable when presented with an array of music choices. Musical preference may remain static or exhibit fluidity, as increased exposure to certain melodies may possibly increase or change preference over time [68]. Non-preferred is deemed least favorable when presented with musical choices. It is worth noting that it could be argued that, by allowing individuals to “choose” their non-preferred music, there is still some level of risk for “preference” bias, even to unfavorable music. Thus, many studies will categorize music on some criteria (i.e., genre, tempo, etc.) and then researchers will randomly choice the specific non-preferred music based off that criteria in order to eliminate any possible “preference” [12,14,16]. Music preference has been shown to repeatedly mediate music benefits while responding to and performing exercise. For the context of discussion in this review, this has been shown in endurance [8,9], anaerobic sprint [16], and resistance exercise modes [12,13,14,15]. Various timing strategies (i.e., during exercise, pre-exercise, warm-up, etc.) have been studied, where music may be played during or before exercise and may change efficacy of music.

**Figure 2:**
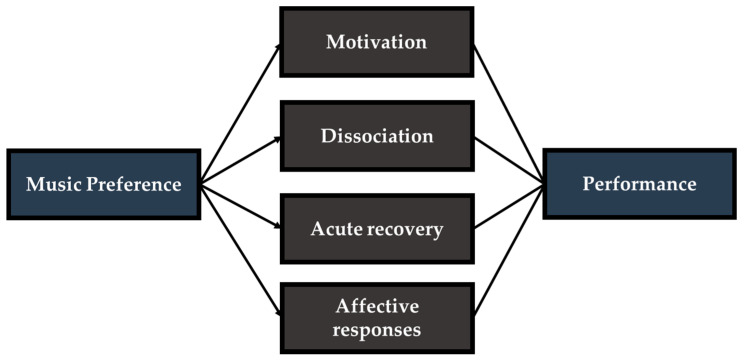
The majority of evidence has suggested that music preference influences exercise performance via the following mechanisms: motivation, dissociation (lower RPE), acute recovery, and affective responses. Interactions between both psychological and physiological mechanisms are responsible for ergogenic effects. Furthermore, preferred and non-preferred music may mediate performance through a single factor listed above or through multiple mechanisms which complement one another.

Although initial investigations have reported a higher efficacy of preferred music, the mechanisms by which preferred music imposes a greater benefit are still being elucidated. Changes in the exercise response with varying music preference appear to be pleiotropic in nature and can be psychological, physiological, and/or psychophysiological (see Figure 2). This section of the review will describe current evidence in these areas in various modes of exercise and interventional timing of preferred and non-preferred music (see Table 1).

### 3.1. Endurance Exercise

Endurance exercise is heavily reliant on being able to repeatedly sustain muscular force and combat fatigue. While music has been widely shown to improve endurance exercise performance, music preference may determine the efficacy of performance enhancement and psychological responses to exercise, although not all studies have shown benefits. In particular, music preference and responses during endurance exercise are the most well described of all modes of exercise. Dyrlund et al. investigated the effects of listening to preferred and non-preferred music during exercise on psychological responses to physical exertion [69]. Two hundred total participants, including both males and females, ran/walked at low, moderate, or high intensities for 20 min. They reported that changes in RPE were not different among conditions, although there was a trend towards lower RPE with preferred music. However, enjoyment and interest in exercise were greater with preferred music compared to no music and non-preferred music. These differences were only apparent in individuals who had the most attentional focus on the music during exercise. This would suggest that benefits of preferred music are contingent on the participant directing their attention to the music versus the task. While the sample size was large, a limitation to this investigation was the between groups study design. Responses to music are highly individualized based off many factors, including gender, age, and intrinsic/extrinsic motivation [11]. Thus, it may have been difficult to compare performance while listening to music between individuals in this case. However, Nakamura et al. followed this by subjecting healthy males to cycling to failure at critical power intensity while listening to no music, and preferred, or non-preferred music [8]. Importantly, a crossover design was used and showed that, although heart rate was not different between conditions, RPE was higher during the non-preferred condition compared to both no music and preferred music. Furthermore, distance cycled was higher while listening to preferred versus non-preferred music. This suggests that non-preferred music results in worsened dissociative ability, even beyond that of no music at all, and that preferred music may be optimal for performance. Further studies have supported these findings, showing that preferred music increases the ability to disassociate and lowers RPE during endurance exercise [70].

The precise mechanisms for this phenomenon are not fully understood, but it appears that preferred music has a greater ability to divert attentional focus away from the discomfort of exercise to the external music stimuli. Previous evidence has shown that greater attentional focus to music during exercise results in lower RPE [69]. Moreover, listening to preferred music has been shown to increase pain tolerance compared to other distracting interventions including cognitive tasks and humor [71]. Overall, this suggests that a primary mechanism of beneficial effects of preferred music is through modulation of attentional focus allowing for focus on the external stimuli of music over discomfort experienced during exhausting exercise.

Interestingly, there have been multiple studies suggesting that benefits of preferred music during exercise may be sex dependent. It has been previously described that males and females may respond to music during exercise incongruously [11,72,73]. However, how music preference may affect males and females differently has been relatively understudied. Cole et al. had competitive male and female runners complete a 12 min Cooper run test while listening to no music, preferred, or non-preferred music [74]. Females had higher running distances while listening to preferred music while male performance did not change regardless of condition. Furthermore, Rasterio et al. recently showed that females has increased heart rate, RPE, and time to exhaustion during an incremental running test while listening to preferred music compared to no music [75]. However, males did not see any apparent benefit from listening to preferred music. Authors did not delineate any particular mechanisms responsible for these sex differences. However, the apparent benefits in females may be due to underlying neuropsychological differences between sexes [76,77]. Previous evidence has shown that females may exhibit higher emotional sensitivity to musical stimuli compared to males [76]. Likewise, neural imaging studies have shown that females listening to music have differing prefrontal cortex activation and superior ability to divert attention from negative thoughts compared to males [77]. While this remains largely speculative at this point in time, sex differences in brain activation during exercise while listening to preferred music may allow females to disassociate from physical and psychophysiological fatigue to a greater degree than males. Sex differences in exercise responses while listening to music, especially in the context of music preference, is unclear and will necessitate further investigation to precisely identify mechanisms responsible for discrepancies.

From a physiological perspective, there is limited evidence to suggest how music preference changes the aerobic exercise response. Many of the observations have been on exercise heart rate and have shown mixed results. Nakamura et al. showed no differences in heart rate between no music, preferred, or non-preferred music during an endurance cycling test [8]. However, it is worth noting that these findings were in healthy males. Others have shown sex-dependent changes in exercise heart rate while listening to preferred music whereby females have higher heart rates compared to male counterparts [75]. However, to complicate interpretations further, females showed no changes in aerobic performance with concurrent increases in heart rate with music making the largest impact past the anaerobic threshold. Females have been shown to have lower sympatho-adrenal responses during exercise compared to males which could attenuate increases in heart rate [78]. Vascular function and vasodilatory ability have also been documented to be higher in females versus males [79]. Documented physiological reasons for differences in exercise heart rate between sexes are counterintuitive in that while, physiologically, one would expect females to perhaps have lower heart rate responses, as previously described, evidence suggests the opposite to be true while listening to preferred music. A likely explanation for this is that females have been suggested to be more susceptible to performance enhancements with preferred music than males while listening to preferred music and increases in heart rate may simply be due to higher workloads. This is supported by recent evidence from my lab showing that females were able to maintain power output better than males while listening to self-selected music during high intensity exercise [11].

Despite this, more clear evidence has suggested that preferred music may induce acute autonomic changes following exercise which may be important in recovery. Archana et al. investigated how listening to preferred music following moderate intensity exercise influenced heart rate variability (HRV) [80]. HRV was significantly lower following exercise while listening to preferred music compared to exercise alone, which suggests increases in parasympathetic output. This is further supported by Jia et al., who showed that an individual listening to their favorite music after cycling resulted in greater activity of the parasympathetic indexes following exercise [53]. Taken together, these findings suggest that the modulation of recovery and cardiac stress following exercise, while listening to music, may be dependent on music preference. Indeed, modulation of stress levels by music, and whether music is sedative or stimulative, has been shown to be dependent on music preference [81]. More research in this area is warranted, but current evidence suggests a potential for preferred music to enhance acute recovery following exercise, which could influence subsequent bouts of exercise or long-term training.

The effects of music preference on blood biomarkers of fatigue during endurance exercise have been studied by one group. Jebabli et al. showed that listening to preferred music resulted in faster running speed and further distance traveled during a 6 min running test [82]. Despite the increases in performance, blood lactate levels measured three minutes after exercise were significantly lower in the music trial compared to no music. Reasons for this remain largely unknown. However, authors speculated that the music may have been relaxing which could reduce muscle tension leading to increases blood flow and lactate clearance. While interesting, these mechanisms remain largely speculative and to date no one has directly measured blood flow changes following exercise with music preference. However, previously mentioned investigations showing autonomic modulation following exercise while listening to preferred music could support the idea of possible vasodilatory effects. Despite the unknowns, the possible increases in muscle blood flow and lactate clearance further support the possible use of preferred music to enhance acute recovery. Systematic approaches to finding physiological underpinnings of preferred music benefits are a dire need, especially in areas of metabolism and neural activation, which may be particularly susceptible to changes while listening to music.

### 3.2. Anaerobic and Sprint Exercise

Overall, the effects of music preference on anaerobic exercise are the least understood. Rasteiro et al. investigated whether listening to preferred music could induce changes in anaerobic threshold intensity [75]. In both males and females, listening to preferred music failed to increase anaerobic threshold intensity, although females were able to perform better at higher intensities. However, the exercise protocol used was an incremental running test, which is not in itself considered solely anaerobic. To date, only one investigation has described how music preference influences true anaerobic sprint exercise performance. Recently, my group studied how listening to preferred or non-preferred music influenced repeated Wingate anaerobic test (WAnT) performance, which is a maximal sprint test on a cycle ergometer [16]. More specifically, 14 physically active males completed 3 × 15 s modified WAnTs with two minutes of active recovery between. During exercise, participants listened to either preferred or non-preferred music. Subjective measures of RPE and motivation were assessed after each WAnT. None of the performance variables (i.e., power output, anaerobic capacity) differed between the music conditions. However, RPE was significantly lower, and motivation was higher while listening to preferred music versus non-preferred. We attributed the lack of differences in performance to the possible standardization of tempo for the music. Previous evidence has shown that tempos >120 bpm can be considered “stimulative” for exercise performance and both preferred and non-preferred music met these tempo criteria [45]. Furthermore, music in itself may have less of an effect on sprint performance due to the maximal nature of the exercise. Since WAnTs require all-out pedaling effort, pacing movement to music is often not possible. The synchrony of movement during exercise has been well established to benefit performance and exercise efficiency [21]. Furthermore, participants are specifically instructed to pedal as fast and as hard as possible during WAnTs, which may also decrease the effectiveness of the music intervention. Indeed, previous evidence has suggested that individuals who are intentional and instructed to exercise to the tempo of music they are listening to are better at movement synchrony [83]. Other music studies outside the context of preference have also documented no changes in sprint performance [23,24]. Although studies on anaerobic performance music preference are limited, at this time it appears that music preference has little to negligible ergogenic effect. Despite the negative findings on performance, music preference appears to have potent effects on RPE and motivation. When paired with the lack of changes in anaerobic capacity, this would suggest that music preference may still influence psychological and psychophysiological measures, even in the absence of performance enhancement. Thus, music preference may have in important role in physical capacity. Previous investigations have noted that increased motivation levels have the ability to increase exercise capacity, even without alterations in maximal physiological variables during exercise [84]. But, my group has recently found that preferred warm-up music results in some benefits to sprint performance and motivation in female collegiate athletes, although the reason for disparities between current and previous findings are unclear (unpublished data). Although data are extremely limited and much more study is needed to form sound conclusions, evidence to date suggest music preference has minimal impact on anaerobic performance but may induce beneficial psychological changes, which could aid in enduring difficult exercise.

### 3.3. Resistance Exercise

Music is a widely used training tool by strength competitors and competitive athletes in the field. Various studies have confirmed the ability of music to impart the enhancement of strength, repetition volume, and the attenuation of fatigue during resistance exercise, although some conflicting information exists [18,85,86,87]. Recent evidence has suggested that disparities between findings may be due to differences in music preference. Indeed, multiple groups have described how music preference influences both upper and lower body resistance exercise performance. De Abreu Araújo et al. subjected 20 young participants to bicep curl and knee extensor exercises at 80% intensity for 10 repetitions, estimated at 1-RM (one repetition maximum) [88]. Participants completed repetitions to failure for each of the exercises while listening to preferred or no music. On average, participants listening to preferred music increased total repetitions by +4 reps (~22%) showing remarkable increases in strength-endurance. While intriguing, the exercises used only small muscle groups and single joint exercises, which may not translate as well to real-world strength. Furthermore, whether detrimental effects may be seen while listening to music one dislikes was not elucidated. In 2018, my group investigated how listening to preferred and non-preferred music during bench press exercises influences performance [14]. Twelve resistance-trained males completed a single set of repetitions to failure at 75% of their 1-RM during explosive bench press. To determine explosive performance, a linear position transducer was used to monitor barbell velocity and power output during the first three reps of the set. Motivation to exercise was also obtained following exercise. Listening to preferred music resulted in increased velocity, power output, repetitions to failure, and subjective motivation compared to non-preferred music. The large magnitude of increased motivation with preferred music suggests that a key underpinning mechanism of performance enhancement is extrinsic motivation with preferred music which likely leads to greater effort, thereby improving explosive performance. However, a major limitation of this investigation is that almost all participants selected the same genre for their preferred (92% of participants chose Rap/Hip-hop) and non-preferred (83% of participants chose Country) music conditions, leaving it unknown if the genre itself is what resulted in performance improvements.

Silva et al. followed this up with a study investigating how preferred and non-preferred music genres influenced grip strength and muscular strength endurance [89]. Participants were allowed to choose preferred and non-preferred music based on genre alone, and researchers selected songs from each. The findings showed that grip strength and lat-pulldown repetition volume increased while listening to preferred music genre. Furthermore, RPE was significantly lower during the preferred music condition. Authors speculated that improvements may have been manifested in the “bottleneck hypothesis” of sensory input, whereby only a finite amount of sensory information reaches the central nervous system at one point in time [90]. In theory, this may cause the high amounts of favorable stimuli from the preferred genre to attenuate the perception of signals from effort and discomfort. This could explain the increased dissociation and repetition volume while listening to preferred versus non-preferred genre music. Improvements in strength could also be explained by this, as diversion of focus on external factors has been linked to improved ballistic exercise performance [91]. While genre preference appears to be a pivotal factor in the efficacy of music-induced improvements of resistance exercise performance, genre is not the sole factor of music, which individuals may prefer. For example, others have shown the music tempo and volume preference may influence exercise responses with listening to music during exercise. Preference for both high tempo and loud volume music have been shown to be increased during high intensity exercise [73,92]. However, it is currently unknown how these might influence resistance exercise performance and future research should focus on determining the specific attributes of music which individuals may exhibit preference for which could improve strength and strength-endurance measures.

### 3.4. Pre-Task/Warm-up Music and Performance

A large barrier for the application of music research to the field and sport is the timing of intervention. Many sports and contests do not allow for the listening of music during competitions, leaving music studies which intervene with listening during exercise of limited value. In these circumstances, listening to music immediately prior to giving effort (i.e., pre-task) or during a warm-up may be more applicable and practical. Both pre-task and warm-up music have been shown to improve performance in a variety of exercise modes, including endurance and resistance-based exercise [42,93]. However, the influence that preference may have on pre-task or warm-up music has only recently been studied. Recently, my group studied the effects of warm-up music preference on rowing exercise performance [9]. Twelve physically active individuals listened to either no music, preferred, or non-preferred music while completing a rowing warm-up at 50% of HR_max_ for 5 min. Following this, music ceased, and participants completed a 2000 m rowing time trial. Preferred warm-up music resulted in higher power output, faster time to completion, and increased motivation. However, RPE remained unchanged regardless of music. Similar ergogenic results with preferred music have been shown in resistance exercise. My group also recently investigated how preferred and non-preferred warm-up influenced repeated bench press performance [12]. Ten resistance-trained males completed a standardized bench press warm-up under preferred and non-preferred music conditions. Participants then completed 2 sets × RTF at 75% bench press 1-RM. While barbell velocity was unchanged, listening to preferred warm-up music resulted in increased repetition volume and motivation across both sets. RPE was unaffected by preference. Still, my group has also shown similar performance enhancement with preferred pre-task music during resistance exercise [13]. Participants completed explosive bench press repetitions to failure at 75% of 1-RM while listening to self-selected preferred music or no music immediately prior to giving effort. Preferred pre-task music resulted in increased barbell velocity, power, repetition volume, and motivation.

Taken together, preferred music appears to maintain ergogenic efficacy even if the intervention is not played during the actual exercise bout. Improvements may be due to differing mechanisms that other investigations employing music interventions during exercise. For example, a widely described ergogenic mechanism of preferred music during exercise is improved dissociation resulting in lower RPE. However, preferred music interventions played solely during a warm-up have shown no changes in RPE [9,12]. Music shifts attention as an external factor which can distract from discomfort or fatigue [44]. Since music during pre-task and warm-up interventions is stopped prior to exercise, this shift in attentional focus is likely lost. It is not currently known what the threshold timing is for warm-up/pre-task music to maintain its efficacy and is an important consideration which needs to be studied in future investigations. However, preferred pre-task and warm-up music still potently increase motivation which is likely to be a key underpinning mechanism for preferred music efficacy even if solely played prior to exercise. While speculative, preferred music may also induce “priming” or stimulation of neural activity. Indeed, warm-up music has been shown to induce increases in plasma catecholamines [54]. Preferred music has also been reported to alter brain activity and pain perception [94,95]. The physiological basis for how preferred music influences responses to exercise are poorly described and understanding these will be paramount in increasing our understanding of how to use music preference to optimize performance.

## 4. Practical Applications and Conclusions

Music provides a very practical means for which to improve acute exercise performance. Music is easily obtainable, cost-effective, and potent as an ergogenic intervention. It is important to the current review to note that music can be easily personalized and individualized. This allows for athletes and coaches to fine tune music interventions on situational or characteristic bases. In the field, music is very commonly listened to during exercise or recovery, similarly by both competitive and recreational athletes. While much evidence supports the use of listening to preferred music during exercise as an ergogenic intervention, this may not be feasible for many sports and athletes. Recent data have now shown that preferred music still retains ergogenic potential even if the music stimulus is only applied prior to exercise or during a warm-up. This has important pragmatic implications as music use across sports and exercise continues to be heightened. Ultimately, the current evidence reviewed suggests that an important aspect to increasing performance with music is personal choice. In many gyms, locker rooms, and competition settings, music is played over a community speaker. Current available data suggest that, if the music played over the speakers is not preferred by the individual giving effort, performance may suffer. Thus, coaches and athletes should consider individual music preferences when attempting to optimize performance and training. While simple, this modification to training or competition music protocols may ultimately lead to potent performance enhancing effects for low amounts of effort and commitment by the athlete or coach. The most feasible approach to achieve this is listening to music using headphones, but other strategies, such as separating athletes into distinct groups by music preference or using smaller speakers at training stations, may work to achieve similar results. While mechanisms for improvements with preferred music are not fully understood, changes in motivation and effort likely play a pivotal role in music benefits during exercise. Future research should aim to dissect the precise physiological and psychological mechanisms responsible for ergogenic effects of preferred music and how these mechanisms may interact with one another to result in a cumulative performance enhancing effect. However, coaches, athletes, and practitioners should attempt to ensure personalized music preference is achieved during activities involving high amounts of effort, which ultimately may result in superior performance.

## Figures and Tables

**Table 1:** Reviewed studies on how music preference influences the exercise response and performance. Conditions, when the music intervention was applied (timing of music), type/mode of exercise (exercise), and primary findings from each investigation are presented. ↑ indicates an increase, ↓ a decrease, ↔ no change in the outcome. RPE = rate of perceived exertion, HRV = heart rate variability RTF = repetitions to failure, HR = heart rate, [La-] = lactate concentration.

Study	Conditions	Timing of Music	Exercise	Primary Findings
Dyrlund et al. (2008)	No music, Preferred, Non-preferred	During exercise	Treadmill running	↑ enjoyment, ↔ RPE (trend towards sig.)
Nakamura et al. (2010)	No music, Preferred, Non-preferred	During exercise	Cycling	↑ cycling distance; ↓RPE; ↔ HR
Connon et al. (2011)	No music, Preferred genre	During exercise	Cycling	↔ performance ↓RPE
Cole et al. (2015)	No music, Preferred, Non-preferred	During exercise	12 min Cooper Running Test	↑ distance run (females), ↔ distance run (males)
Archana et al. (2016)	No music, Preferred	Post Exercise	Cycling	↓ low frequency/high frequency components of HRV
Ballmann et al. (2018)	Preferred, Non-preferred	During exercise	Bench press	↑ barbell velocity; ↑ power; ↑ RTF; ↑ motivation
de Abreu Araújo et al. (2018)	No music, Preferred	During exercise	Bicep curl, Knee Extension	↑ repetitions to failure
Ballmann et al. (2019)	Preferred, Non-preferred	During exercise	Wingate sprints	↔ performance ↓RPE; ↑ motivation
Karow et al. (2020)	No music, Preferred, Non-preferred	Warm-up	Rowing	↑ power output; ↓ time; ↑ motivation; ↔ RPE
Jebabli et al. (2020)	No music, Preferred	During exercise	6 min Run Test	↑ running speed; ↑ distance covered; ↓ Blood [La-] ↔RPE
Rasteiro et al. (2020)	No music, Preferred	During exercise	Incremental Running Test	↑ HR (females); ↑ RPE (females); ↑ time (females) ↔ outcomes (males)
Silva et al. (2020)	No music, Preferred genre, Non-preferred genre	During exercise	Hand grip, Lat-pull down	↑ RTF; ↑ grip strength; ↓RPE
Ballmann et al. (2021)	No music, Preferred	Pre-task	Bench press	↑ barbell velocity and power; ↑ RTF; ↑ motivation
Ballmann et al. (2021)	Preferred, Non-preferred	Warm-up	Bench press	↑ RTF; ↔ barbell velocity; ↑ motivation; ↔RPE

## Data Availability

Not applicable.
